# Respiratory Tract Infection Leading to the Diagnosis of Hereditary Hemorrhagic Telangiectasia in a Puerto Rican Patient: A Case Report

**DOI:** 10.7759/cureus.67638

**Published:** 2024-08-23

**Authors:** Felix Rivera Troia, Milaris M Sanchez-Cordero, Fernando J Ocasio Villa, Lara Diez Asad

**Affiliations:** 1 Surgery, University of Medicine & Health Science, Bassettiere, KNA; 2 Genetics, Ponce Health Sciences University, Ponce, PRI; 3 Internal Medicine, Mayaguez Medical Center, Mayaguez, PRI; 4 Genetics, Western Oncology Cancer Center, Mayaguez Medical Center, Mayaguez, PRI; 5 Dermatology, Ponce Health Sciences University, Ponce, PRI

**Keywords:** av malformation, hispanic population, puerto rico, olser weber rendu, hereditary hemorrhagic telangiectasis

## Abstract

Hereditary hemorrhagic telangiectasia (HHT), also known as Olser-Weber-Rendu, is a genetic disorder characterized by abnormal blood vessel formations. Inherited in an autosomal dominant pattern, the condition manifests through symptoms such as nosebleeds (epistaxis), spider-like blood vessels (telangiectasias) in mucous membranes often appearing as spots or lesions, and abnormal connections between arteries and veins. Here we present the case of a 66-year-old male who came to the emergency department (ED) with symptoms consistent with a respiratory tract infection. Relevant lab tests revealed a pattern of iron deficiency anemia, leading to a referral for management. Further investigation uncovered a long history of spontaneous nosebleeds, prompting a referral for genetic sequencing and analysis in the hopes of ruling out bleeding disorders. The test results were positive for a heterozygous pathogenic variant in the ACVRL1 gene, a gene associated with HHT and pulmonary arterial hypertension (PAH). Hereditary hemorrhagic telangiectasia is considered a relatively common disorder; however, there is a notable lack of comprehensive data regarding its prevalence within the Puerto Rican and broader Hispanic populations. This knowledge gap is significant because it hampers the ability to understand the full scope of the condition's impact and how it might present differently in diverse genetic backgrounds. Our goal is to enhance the understanding of HHT's prevalence and manifestations in these populations. This contribution is intended to support and stimulate further research, which could lead to more accurate epidemiological data, improved diagnosis, and a better understanding of the condition.

## Introduction

Hereditary hemorrhagic telangiectasia (HHT), also referred to as Osler-Weber-Rendu syndrome, is an autosomal dominant disorder marked by the presence of epistaxis, telangiectasia, and arteriovenous malformations (AVMs) affecting the skin and mucous membranes. It is a relatively common but often underrecognized disorder that affects all ethnic and racial groups, with a prevalence of one in 5,000 people [1]. The most common presenting symptom is spontaneous recurrent epistaxis [2]. Other symptoms associated with the disease include iron deficiency anemia (IDA), shortness of breath, headaches, and seizures [3].

Hereditary hemorrhagic telangiectasia is associated with many subtypes based on the gene mutated. Type 1 HHT results from mutations in ENG found on chromosome 9, a gene that encodes endoglin, a protein involved in TGF-ß-mediated cell signaling and angiogenesis suppression [4]. Type 2 HHT occurs due to a mutation in ACVRL1 on chromosome 12, a gene in charge of regulation of endothelial cell proliferation and arterial identity during angiogenesis [5]. Furthermore, HHT associated with juvenile polyposis results from mutations in the SMAD4 gene located on chromosome 18. Of these, by far the most common is the ENG gene mutation, found in up to 61% of patients diagnosed with HHT [6].

Treatment of the disease is based primarily on controlling bleeds. Nasal packing, locally applied pressure, and the use of electrocauterization are often implemented in the management of epistaxis depending on the severity of symptoms [7]. Iron replacement therapy and surgical resection are sometimes invoked when managing gastrointestinal bleeds. However, due to the systemic nature of the disease, a multidisciplinary care approach involving different specialties is often required to successfully manage treatment [8].

## Case presentation

A 66-year-old man presented to the emergency department (ED) with complaints of shortness of breath, productive cough, and fever that had been ongoing for the past three days. Hematologic and biochemical testing revealed low hemoglobin concentration, elevated red blood cell distribution width (RDW), and low serum iron and ferritin levels, consistent with IDA (Table 1). The patient was treated for his respiratory symptoms accordingly and was subsequently referred to the clinic for management of his IDA. Further investigation revealed that the patient had a long history of spontaneous, recurrent nosebleeds and that his sibling, as well as other family members on his mother’s side, also experienced similar issues. These findings prompted a referral for genetic testing/sequencing in hopes of ruling out bleeding disorders. Genetic sequence/analysis further revealed a pathogenic variant identified in the ACVRL1 gene, which confirmed a diagnosis of HHT (Table 2). 

**Table 1:** Hematological (CBC) and biochemical test results

Test	Results	Reference range
White blood cell (WBC) count	6.63	3.98-10.04x10^3/uL
Red blood cell (RBC) count	4.32	4.63-6.08x10^6/uL
Hemoglobin (HBG)	9.9	13.7-17.5 g/dL
Hematocrit (HCT)	32.9	40.1-51.0%
Mean corpuscular volume (MCV)	76.2	79.0-94.8 fL
Mean corpuscular hemoglobin (MCH)	22.9	25.6-32.2 pg
Mean corpuscular hemoglobin concentration (MCHC)	30.1	32.2-36.5 g/dL
Red cell distribution width (RDW) standard deviation	46.8	35.1-46.3 fL
RDW coefficient of variation	17.2	11.6-14.4%
Platelet count	168	163-269 10^3/uL
Ferritin	15.8	30-400 ng/mL
Iron	20	50-150mcg/dL
Total iron binding capacity (TIBC)	328	240-450mcg/dL
Iron saturation	6	20-50%

**Table 2:** Genetic sequencing/analysis shows a positive result for the ACVRL1 gene mutation

Gene	Variant	Zygosity	Variant classification
ACVRL1	c.1121G>A (p.Arg374Gln)	Heterozygous	Pathogenic

The patient's medical history included systemic hypertension and pulmonary hypertension, both of which were pertinent to his current health status. He had reported issues with medication adherence for managing his blood pressure. Notably, the patient did not consume alcohol and maintained a non-smoking lifestyle. On the physical exam, it was evident that the patient presented with red-spotted lesions on his tongue; however, none were noticed on the lips or other areas of his body.

The patient was informed and oriented about his condition accordingly. He was initially receiving Venofer 200 mg in 10 mL of saline to manage low serum iron levels. However, as he stopped responding to this treatment, he has since been switched to ferrous sulfate therapy, which he is currently tolerating well.

## Discussion

Hereditary hemorrhagic telangiectasia is considered the second most prevalent inherited bleeding disorder, affecting approximately one in every 5,000 people [9, 10]. The disease is inherited in an autosomal dominant pattern and is characterized by the presence of recurrent nosebleeds, malformed vessels that appear on the skin or mucous membranes (telangiectasias), and abnormal AVMs. Estimates suggest that 1% to 10% of patients with HHT develop cerebral AVMs, while 15% to 45% develop pulmonary AVMs. Strokes, seizures, and migraines are considered complications that arise from these vascular malformations. Although AVMs are less common than other causes of stroke, they are significant because they can be identified and treated in high-risk patients before complications arise [11].

Hereditary hemorrhagic telangiectasia is an often underdiagnosed bleeding disorder, with only 10% of those affected receiving a confirmed diagnosis. The Curaçao diagnostic criteria are used to aid in the diagnosis of this disorder based on the presenting clinical signs and symptoms (Table 3). A definitive diagnosis of HHT is considered for those who meet three or more criteria; those who meet at least two criteria are considered to possibly have HHT, and patients meeting less than two criteria are unlikely to have the disease [12]. One study published its use in the pediatric population and presented its reliability in diagnosing children who meet three or four of the diagnostic criteria [13].

**Table 3:** Curaçao criteria Definite HHT: if three criteria are present; possible HHT: if two criteria are present; unlikely HHT: if fewer than two criteria are present AVM: arteriovenous malformation; HHT: hereditary hemorrhagic telangiectasia

Criteria	Characteristics
Epistaxis	Spontaneous, recurrent nosebleeds
Telangiectasia	Multiple at characteristic sites (lips, oral cavity, nose, fingers)
AVM	Any of the following: cerebral AVM, spinal AVM, pulmonary AVM, hepatic AVM, gastrointestinal telangiectasia (with or without bleeding)
Family history	A first-degree relative with HHT according to the criteria

While HHT impacts individuals across all racial groups, there has been limited research into racial disparities among patients with the condition. One study conducted a retrospective chart review of patients diagnosed with HHT from 2014 to 2022 [14]. Their review included patients from Asian, African American, Hispanic/Latino, and White/Caucasian populations. Their results showed that patients who identified as Asian experienced an increased risk of pulmonary AVMs compared to the White population, and those who identified as Hispanic/Latino had an increased risk of developing cerebral AVMs when compared to the White population. Both results were statistically significant (P = 0.03 and P<0.01, respectively [14]. A separate study examining the epidemiology of HHT in the United States, which analyzed data from 2013 to 2017, discovered that the prevalence of the disease had risen from 6.1 to 12.1 per 100,000 people. The most significant increases were observed in patients aged 18-29 years of age and those who were 60 years or older [15]. Few studies report the prevalence of the disease in the Hispanic/Latino population specifically; however, a cross-sectional study conducted in Buenos Aires, Argentina, reported that the prevalence of HHT was 3.2 out of every 10,000 people. The study included patients from a specific healthcare system in Argentina who were 18 years of age or older and had a confirmed diagnosis of HHT. Furthermore, they found that the disease was almost twice as common in females compared to males and that the average age was approximately 55 years for both [16].

## Conclusions

This case report highlights the importance of recognizing HHT in patients presenting with recurrent nosebleeds and iron deficiency anemia, especially when there is a family history of similar symptoms. The diagnosis of HHT in this 66-year-old male was confirmed through genetic testing, revealing a pathogenic variant in the ACVRL1 gene. While HHT is a well-documented disorder, there remains a significant gap in understanding its prevalence and manifestations within Puerto Rican and broader Hispanic populations. Addressing this gap is crucial for improving our understanding of the condition and developing effective diagnostic methods. By presenting this case within the Puerto Rican and Hispanic communities, we aim to stimulate further research, which could lead to more accurate epidemiological data and enhanced diagnostic approaches.
